# Structural and Chemical Profiling of the Human Cytosolic Sulfotransferases 

**DOI:** 10.1371/journal.pbio.0050097

**Published:** 2007-04-10

**Authors:** Abdellah Allali-Hassani, Patricia W Pan, Ludmila Dombrovski, Rafael Najmanovich, Wolfram Tempel, Aiping Dong, Peter Loppnau, Fernando Martin, Janet Thonton, Aled M Edwards, Alexey Bochkarev, Alexander N Plotnikov, Masoud Vedadi, Cheryl H Arrowsmith

**Affiliations:** 1 Structural Genomics Consortium, University of Toronto, Toronto, Ontario, Canada; 2 Department of Medical Biophysics, University of Toronto, Toronto, Ontario, Canada; 3 European Bioinformatics Institute, Cambridge, United Kingdom; 4 Banting and Best Department of Medical Research, University of Toronto, Toronto, Ontario, Canada; 5 Department of Physiology, University of Toronto, Toronto, Ontario, Canada; Brandeis University, United States of America

## Abstract

The human cytosolic sulfotransfases (hSULTs) comprise a family of 12 phase II enzymes involved in the metabolism of drugs and hormones, the bioactivation of carcinogens, and the detoxification of xenobiotics. Knowledge of the structural and mechanistic basis of substrate specificity and activity is crucial for understanding steroid and hormone metabolism, drug sensitivity, pharmacogenomics, and response to environmental toxins. We have determined the crystal structures of five hSULTs for which structural information was lacking, and screened nine of the 12 hSULTs for binding and activity toward a panel of potential substrates and inhibitors, revealing unique “chemical fingerprints” for each protein. The family-wide analysis of the screening and structural data provides a comprehensive, high-level view of the determinants of substrate binding, the mechanisms of inhibition by substrates and environmental toxins, and the functions of the orphan family members SULT1C3 and SULT4A1. Evidence is provided for structural “priming” of the enzyme active site by cofactor binding, which influences the spectrum of small molecules that can bind to each enzyme. The data help explain substrate promiscuity in this family and, at the same time, reveal new similarities between hSULT family members that were previously unrecognized by sequence or structure comparison alone.

## Author Summary

We metabolize many hormones, drugs, and bioactive chemicals and toxins from the environment. One family of enzymes that participate in the metabolic process consists of the cytosolic sulfotransferases, or SULTs. SULTs have a variety of mechanisms of action—sometimes they inactivate the biological activity of the chemical (e.g., in the case of estrogen). At other times, the enzymes make the chemical more toxic (e.g., for certain carcinogens). Humans have 12 distinct SULT enzymes. Determining how each of these human enzymes recognizes and distinguishes between the thousands of chemicals we confront each day is essential for understanding hormone regulation, assessing environmental risk, and eventually developing better, more-effective drugs. We have studied the human SULT family of enzymes to profile which small molecules are recognized by each enzyme. We also visualized and compared the detailed structural features that determine which enzyme interacts with which molecule. By studying the entire family, we discovered new ways in which chemicals interact with each enzyme. Furthermore, we identified new inhibitors and inhibitory mechanisms. Finally, we discovered functions for many of the human enzymes that were previously uncharacterized.

## Abstract 3

Structural genomics and substrate screening provide "chemical fingerprints" and insights into substrate promiscuity for the human family of drug- and hormone-metabolizing cytosolic sulfotransferase enzymes.

## Introduction

**Figure 1:**
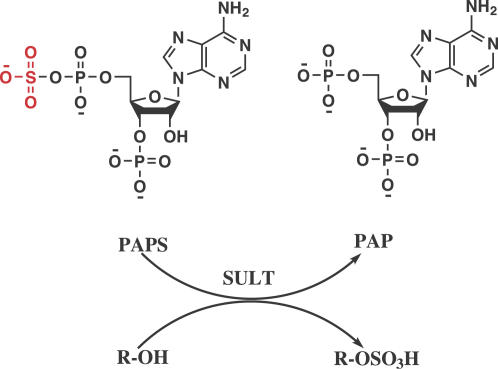
Schematic of the Reaction Catalyzed by SULTs

Cytosolic sulfotransferases (SULTs) comprise a family of enzymes that catalyze the transfer of a sulfonate group from 3′-phosphoadenosine 5′-phosphosulfate (PAPS) to an acceptor group of the substrate (Figure 1). In doing so, SULTs modulate the activities of a large array of small endogenous and foreign chemicals, including drugs, toxic compounds, steroid hormones, and neurotransmitters. Because sulfonated molecules are highly soluble in water and easily excreted from the organism, SULTs are often referred to as enzymes of chemical defence. In some cases, however, SULTs activate certain compounds from food and the environment into mutagenic and carcinogenic metabolites [1].

To date, 13 human cytosolic sulfotransferase (hSULT) genes have been identified; they partition into four families [2,3]: SULT1, SULT2, SULT4, and SULT6. Although the family members share considerable sequence and structural similarity, they appear to have different biological functions. The SULT1 family comprises nine members divided into four subfamilies (1A1, 1A2, 1A3, and 1A4; 1C1, 1C2, and 1C3; 1B1; and 1E1). The SULT1A3 and SULT1A4 genes appear to have arisen from a segmental duplication and encode the same protein [4]. Members of the SULT1 family have been shown to sulfonate simple phenols, estradiol, and thyroid hormones, as well as environmental xenobiotics and drugs. The SULT2 family has two genes, encoding three proteins (SULT2A1, SULT2B1a, and SULT2B1b), which catalyze sulfonation of hydroxyl groups of steroids, such as androsterone, allopregnanolone, and dehydroepiandrosterone (DHEA). SULT4A1 is the only member of the SULT4 family. The fact that it is highly conserved and expressed primarily in the brain suggests an important function; however, no activity or function has been identified for this gene [5]. Finally the SULT6B1 gene is expressed in the testis of primates, but neither the protein nor its enzymatic activity has been characterized [3].

Recent progress in the structural biology and characterization of the catalytic mechanism of hSULTs has established that many family members have distinct, but overlapping, substrate specificities and that the enzymes have a sequential catalytic mechanism that is susceptible to substrate inhibition [6,7]. Nevertheless, only a few of the human enzymes have been subjected to detailed structural and mechanistic studies [6,8–16], and there are no reports of a systematic comparison among all the hSULTs. Understanding the structural and mechanistic basis for specificity among hSULTs is essential to elucidate their role in the metabolism of regulatory hormones, drugs, and carcinogens, and may assist in chemical risk assessment and the design of more-effective therapeutics. Here we report the crystal structures of five of the 12 structurally unique hSULTs. These structures, combined with those previously reported for six other hSULTs, allowed a comprehensive comparison of both global and local structural features. We further screened nine hSULTs for binding activity toward a set of 90 potential substrates and inhibitors, and eight hSULTs for enzymatic activity toward 31 potential substrates in order to better understand the relationship between binding specificity, activity, and structure within the hSULT family. These data, combined with detailed structural analysis of substrate binding sites, reveal relationships between family members not previously apparent from sequence analysis. “Chemical fingerprints” of the spectrum of small molecules that bind in the presence and absence of the cofactor product, 3′-phosphoadenosine 5′-phosphate (PAP), demonstrate a marked change in the small molecule binding profile upon PAP binding. This result, combined with the structural data, suggests PAPS has a strong influence on which compounds may bind in the substrate binding site and raises the possibility that the enzymes might be inhibited by chemically related compounds that are not productive substrates. The binding studies also provide insight into potential functions of the under-characterized SULT1C subfamily and of SULT4A1, an orphan member of the SULT family expressed primarily in the brain.

## Results

### Completion of the Structural Coverage of hSULTs

**Figure 2:**
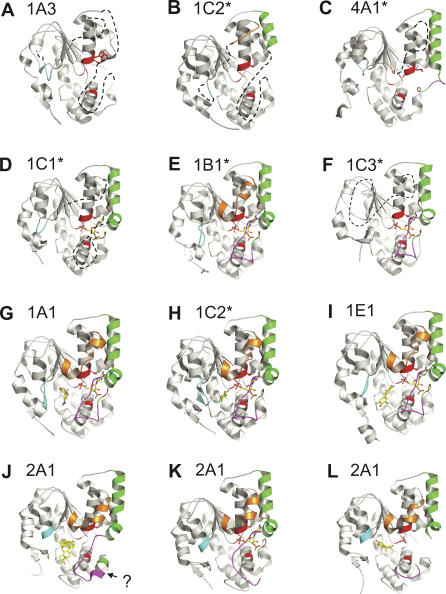
Representative Structures for Each of the hSULTs Grouped According to Similar Global Structural Features Apo proteins with considerable disorder in several loops: (A) SULT1A3 (1CJM), (B) SULT1C2 (2AD1), and (C) SULT4A1 (1ZD1). Structures with increased order due to binding of PAP (green helices, purple loop): (D) SULT1C1 (2ETG), (E) SULT1B1 (1XV1), and (F) SULT1C3 (2H8K). Ternary complexes display further ordering of substrate binding loops: (G) SULT1A1 with PAP and *p*-nitrophenol (1LS6), (H) SULT1C2 with PAP and PCP (2GWH), and (I) 1E1 with PAP and 3,5,3′,5′-tetrachloro-biphenyl-4,4'-diol (1G3M). Structures with unusual features that likely reflect catalytically unproductive proteins: (J) SULT2A1 bound to DHEA, but without PAP (1J99); compare to (K) structure of the same protein with PAP (1EFH), and (L) with androsterone (1OV4). The structures we solved are labelled with an asterisk. The question mark (?) indicates a helix formation, which leads to a non-productive conformation. The Protein Data Bank code for each structure is shown in parentheses. The proteins are represented by a ribbon model; PAP and substrate are shown as stick models coloured per element (carbon in yellow, oxygen in red, nitrogen in blue, and phosphate in magenta). The loops are coloured as discussed in the text (gold, cyan, green, and purple).

**Figure 3:**
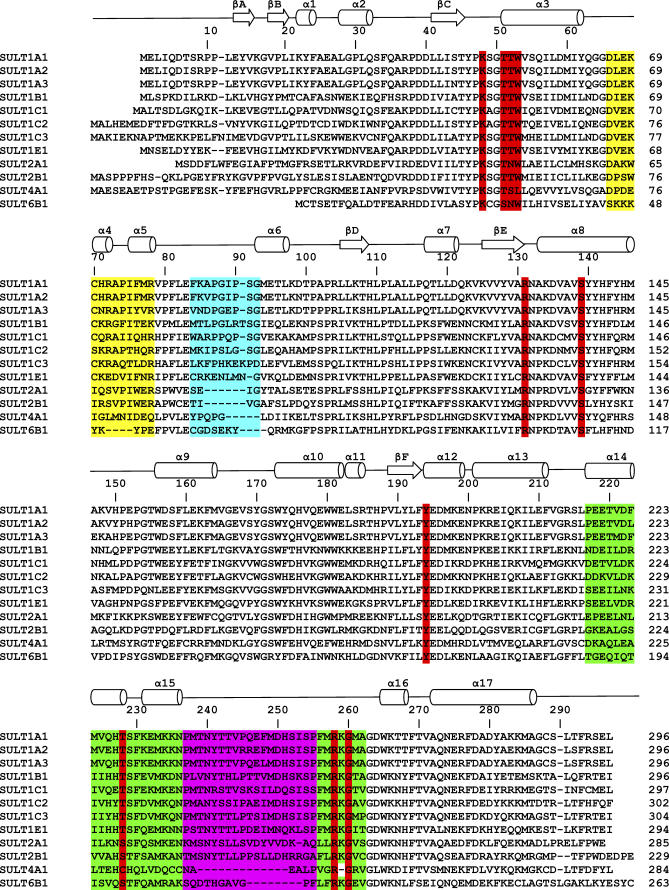
Sequence Alignment of hSULTs Showing Structural Features with Colour Coding Corresponding to That of the Structures in Figure 2

The crystal structures of SULT1C3 bound to PAP, apo SULT1C2, a ternary complex of SULT1C2 bound to PAP, and the environmental toxin, pentachlorophenol (PCP), and SULT4A1 were solved at 3.2, 2.0, 1.8, and 2.2 Å, respectively (Figure S1 and Table S1). We also recently reported the structures of SULT1B1 and SULT1C1 bound to PAP at 2.1 and 1.8 Å, respectively [17]. The structures of a single subunit of each of these normally dimeric proteins are presented in Figure 2 along with a representative structure of each of five other SULT family members previously reported in the literature [8,10–15]. Six additional SULT structures, which are available in the Protein Data Bank, are presented in Figure S2. As expected, all SULTs share the same basic fold: a central four-stranded parallel β-sheet surrounded by α-helices and three loops that are often disordered (dashed lines) in the absence of PAP and/or substrate. These disordered segments comprise a 13-residue loop (shown in gold), a 4~10 residue loop (cyan), and a large 32~46 residue loop (green and magenta). These loops have been mapped onto the aligned protein sequences in Figure 3 using the same colouring scheme. The degree of disorder and the exact conformation of these loops vary considerably across the family, but in general, the presence of ligands (cofactor and/or substrate) is coupled with increased order, namely, the formation of helices α4-α5 (gold), and α14-α15 (green). In some cases, partial stabilization can be attributed to molecular packing in the crystal, as in, for example, the stabilization of α14 (green) in apo SULT1C2. The binding site for PAP or PAPS (PAP(S)) is nearly identical in all structures bound to these ligands, with highly conserved residues contributing to the binding pocket (highlighted in red in Figures 2 and 3). It is interesting to note that the SULT6B1 sequence in the protein databases lacks the N-terminal region, which encodes a β-sheet thought to be an important structural component of the SULT fold (Figure 3). We note that the recombinant SULT6B1 did not express in our attempts to purify it from bacteria.

### Structural Comparison Supports a Role for PAPS in Priming the Conformation of Substrate Binding Loops

It is generally agreed that sulfonation takes place via a sequential mechanism in which a ternary enzyme complex is first formed, followed by reaction and release of products [7]. However, both random and ordered binding of the substrate and cofactor molecules have been reported, and the detailed kinetic mechanism (or mechanisms) of the sulfonate transfer reaction is the subject of continuing research (reviewed in [7]). Comparison of all the available structural data provides insight into the order of substrate and cofactor binding. The structures provide evidence for both binary complexes (enzyme/substrate and enzyme/cofactor) consistent with a random bi-bi mechanism and ruling out an ordered mechanism in which binding of substrate requires binding of cofactor (or vice versa). This is in agreement with a detailed kinetic analysis for SULT1E1 [18]. However, a closer inspection of the structures also suggests that binding of substrates may not be completely uncoupled from binding of the cofactor. In all the structures with the co-factor product, PAP, α14-α15, and the C-terminal segment of the largest flexible loop (green in Figure 2) are ordered. This region contributes three absolutely conserved residues necessary for PAPS binding, T228, R258, and G260 (SULT1A1 numbering and red in Figure 2). Importantly, although the other loops (cyan, gold, and magenta) do not contribute directly to PAPS binding, they are more likely to be partially ordered in the presence of PAP(S). The PAP(S)-induced ordering of α14-α15 and residues 256–262 (green and red) may also restrict the conformations available to the intervening substrate-binding magenta loop when PAP is bound. Thus, the structural data suggest that PAPS binding tends to prime the cyan, gold, and magenta loops for binding to the substrate.

On the other hand, the structure of SULT2A1 bound to androsterone [13] (Figure 2L) hints that binding of substrates does not prime the PAPS binding loops. In this structure, the substrate-binding cyan and gold loops are ordered, but the magenta loop and adjacent PAPS binding residues (green and red portion of the loop) are disordered. Thus, although in this case, substrate and PAP(S) molecules can each bind independently to the enzyme as in a random bi-bi mechanism, there may be some degree of cooperativity between substrate binding and prior cofactor binding, but not vice versa. Given that the estimated cellular concentration of PAPS is well above that of most substrates, this may be relevant to the catalytic mechanism.

The family-wide structural comparison also suggests an additional or alternative explanation for the well-documented substrate inhibitory effect. Previously reported cases of substrate inhibition have been attributed either to two substrate molecules occupying the active site at the same time [8,19] or to the ability of substrates to bind in unproductive orientations at higher concentrations [6,14]. Examination of the structures in Figure 2 suggests a third or alternative mechanism; at high concentrations, substrates may bind in a mode in which the binding loops are incompatible with PAPS binding. This case is exemplified by the structure of SULT2A1 with DHEA [14]. As shown in Figure 2J, this structure can accommodate two substrate orientations at roughly 30° to one another. Comparison with other hSULT structures strongly suggests that SULT2A1 in this structure adopts a non-productive conformation. A portion of the green-and-magenta loop that contributes two residues for PAP(S) binding is folded into a helix, orienting the crucial PAPS binding residues away from the cofactor binding pocket. This helix conformation is not an intrinsic feature of SULT2A1, because in the SULT2A1–PAP complex, this region adopts a conformation similar to that in other SULT–PAP structures (compare Figure 2K and 2J with this region highlighted by a question mark). Thus, it appears that the structure adopted by SULT2A1 with two molecules of DHEA is incompatible with PAP(S) binding and that this conformation is induced by the substrate. This is further evidence of “communication” between the substrate binding site and the PAPS binding site.

### Ligand Binding and Activity Profiles Reveal Enzyme-Specific Chemical Fingerprints

**Figure 4:**
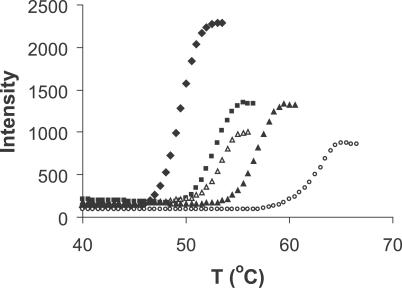
Thermo-denaturation/Aggregation Profile of SULT2A1 The intensity of scattered light is plotted as a function of temperature *(T)* for SULT2A1 in the absence of any ligand (filled diamond), and in the presence of AMP-PNP (filled square), lithocholic acid (open triangle), PAP (filled triangle), and both PAP and dehydroisoandrosterone 3-sulfate (open circle)**.** The inflection point of the denaturation curve is taken as *T*_agg_ as reported in Table 1.

In order to predict and understand the fate of xenobiotics and drug candidates in humans, it is essential to better understand the selectivity and specificity of binding and activity within the hSULT family. Although detailed analyses of individual structures have been very informative in this regard [6,8–12,16], we sought to compare all active sites relative to the spectrum of small molecules that can bind to each site. However, several of the proteins whose structures were solved in this study have not been previously characterized, and it was difficult to directly compare data from the literature due to differences in experimental conditions. Therefore, in order to evaluate specificity and selectivity in a consistent manner, nine purified, recombinant hSULTs were screened for binding to a library of 90 small molecules (Table S2) that comprised known substrates, inhibitors, related hormones, bioamines, and drugs [20,21]. In order to profile the entire hSULT family, we made use of the well-known fact that equilibrium binding of a ligand increases the thermal stability of a protein in a manner proportional to the concentration and binding affinity of the ligand [20,21]. In a multi-well format, the thermal stability of each hSULT was monitored as a function of temperature and in the presence or absence of compounds (Figure 4). In the absence of compounds, well-behaved, sigmoidal thermo-denaturation/aggregation profiles were obtained for hSULTs 1A1, 1A3, 1B1, 1E1, 1C1, 1C2, 1C3, 2A1, and 4A1. SULT2B1b did not denature within the range of temperature used for this type of analysis (up to 80 °C). In this screening format, compounds that stabilize a protein by more than 2° C are scored as positives (Table 1). It was not possible to assay binding to ligands in the presence of the sulfonated co-factor, PAPS, because the sulfonate transfer reaction would have taken place. However, except for SULT4A1, PAPS and PAP had equivalent stabilizing affects on all hSULTs. Thus, PAP was used as a substitute for PAPS in considering the effect of cofactor upon substrate binding, and screens for the binding of ligands were performed in the absence and presence of a saturating amount of PAP.

**Table 1:**
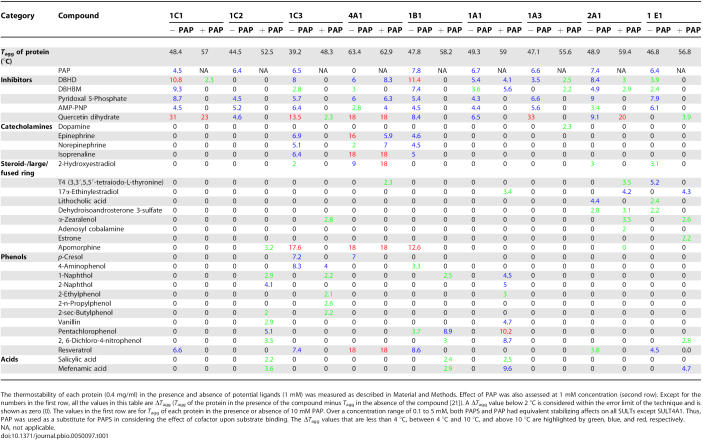
Thermostability Screen for Ligand Binding to hSULTs

Based on these binding results, a set of 20 compounds that bound to at least one hSULT plus 11 additional related compounds or known substrates were used as a pool of potential substrates for enzymatic activity of hSULTs 1A1, 1A3, 1B1, 1C1, 1C2, 1C3, 1E1, and 2A1 (Table 2). We monitored the conversion of PAPS to PAP by high-performance liquid chromatography (HPLC) as a tractable method of screening multiple proteins against multiple substrates (eight proteins and 31 substrates in this study), and the results serve as a convenient first approximation of enzymatic activities. We note that due to the relatively low sensitivity of this method, we were not able to reliably assay substrates at nanomolar concentrations, and therefore, some of the results may be complicated by substrate-mediated inhibition. Indeed, inspection of Table 2 shows that in some cases, the highest levels of activity were observed at lower substrate concentrations. This is especially true of SULT1A1, an enzyme for which significant substrate inhibition has been noted previously [8].

**Table 2:**
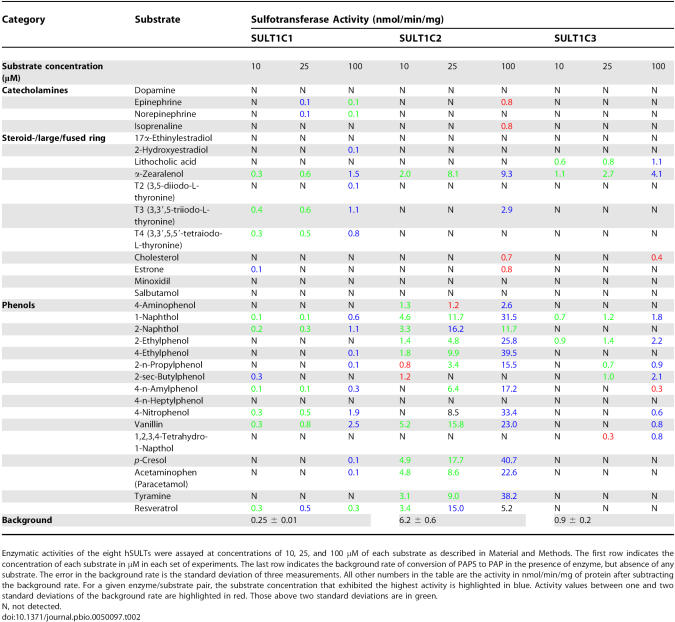
Sulfotransferase Activity Screen of hSULTs

**Table 2:**
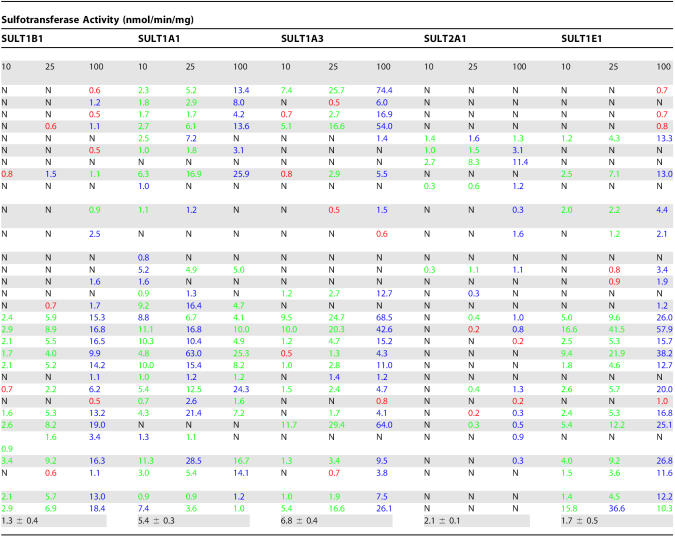
Extended.

The combined ligand binding and activity screens revealed a unique “chemical fingerprint” for each hSULT (Tables 1 and 2). First, as expected from previous studies, there was considerable overlap in the substrate specificity for enzymatic activity. For example, all hSULTs assayed here were able to sulfonate a number of phenolic compounds such as naphthols and/or alkylphenols. However, within each substrate profile, there were also elements of specificity. For example, SULT1A1 and SULT1A3 were the only two hSULTs that showed significant activity toward catecholamines compared to other substrates, with SULT1A3 being more specific for dopamine, as expected from previous studies [22–26]. SULT1A3 was also the only protein to bind dopamine in the binding assays, consistent with its designation as human dopamine sulfotransferase [25]. It is interesting to note that in the past, SULT1A1 and 1A3 have been distinguished from one another in tissue fractions by the higher sensitivity of SULT1A1 to inhibition by 2,6-dichloro-4-nitrophenol (DCNP) [27]. Although we did not measure inhibition by this compound, we note that SULT1A1 bound DCNP in the presence of PAP, whereas SULT1A3 did not (Table 1).

Six hSULTs (1C1, 1C2, 1E1, 1B1, 1A1, and 1A3) had enzymatic activity toward resveratrol, a polyphenolic compound present in grapes and wine, with possible anticancer and cardioprotective activities [28]. The activity profiles for resveratrol also displayed evidence of substrate inhibition by this compound for SULTs 1C1, 1C2, 1A1, and 1E1. Acetaminophen was a substrate for SULTs 1A1, 1E1, 1A3, 1C2, and 1B1, but not SULT1C3 and SULT2A1. Substrates for SULT1C3 have not been reported previously. Our data indicate that this recently identified member of the hSULT family is able to sulfonate *p*-nitrophenol, 1-naphthol, 2-ethylphenol, 2-*n*-propylphenol, and 2-*sec*-butylphenol, as well as the steroid-related compounds, α-zearalenol and lithocholic acid. SULT1C3 appeared to be most active with α-zearalenol (4.1 nmol/min/mg) and 2-ethylphenol (2.2 nmol/min/mg). These data suggest SULT1C3 may contribute to the metabolism of steroid and phenolic compounds. Finally, SULTs 2A1 and 1E1, which are reported to metabolize steroids [29–32], both bound to and sulfonated multiple steroid and steroid-like compounds with different apparent specificities. These data show that despite the limitations of our rapid screening method, the enzymatic activity data in Table 2 reflects, to a first approximation, the expected relative substrate activities reported in the literature and reveal new activities toward pharmacologically important compounds. The binding data, on the other hand, suggest a more complicated situation.

### PAP Is Able to Alter Ligand Binding Profiles

The ligand binding profiles were remarkably different in the presence and absence of PAP (with the exception of SULT4A1, which will be discussed separately below). Some known substrates for the well-characterized SULTs appeared to bind only in the presence of PAP—for example, dopamine for SULT1A3 [24,25], and 1-naphthol for SULT1B1 [33]—whereas many previously unreported compounds bound to these and other family members in the absence of PAP. It is interesting to note that not all known substrates, nor all of those with reactivity in our activity screens, were found to *bind* to the enzyme in the presence of PAP. For example, resveratrol was a substrate for SULT1A3 and SULT1C2, but did not stabilize either protein in the binding assays, either in the presence or absence of PAP. There are several reasons that may account for these observations. First, the enzymatic screen is likely more sensitive than the ligand binding assay in which compounds with K_m_ or K_d_ values in the high micromolar range are less likely to be detected [20,21]. Second, some ternary complexes may not be significantly stabilized relative to the PAP–enzyme complex (especially at elevated temperatures). Finally, the presence of the sulfonate group of PAPS may also contribute to binding of substrates, and these cases may not be detected in our binding assay. Interestingly, binding of SULT1C1, 1B1, and 1E1 to resveratrol was only observed in the absence of PAP, but all are active toward this substrate. Nevertheless, the radically different binding profiles observed in the presence and absence of PAP are consistent with the structure-based mechanisms proposed above. Specifically, PAP (and presumably PAPS) appears to prime the substrate binding loops for subsequent binding to certain substrates, whereas in the absence of cofactor, the loops are free to bind alternative ligands (perhaps only at high concentrations), or non-productive ligand-bound conformations may exist. This priming of the substrate binding loops is likely made possible by flexibility of the binding loops observed in the structure. This structural plasticity may allow a reconfiguration of substrate binding loops in the absence of PAP(S) in order to bind a different chemical class of compound. For example, both SULT1C3 and SULT1B1 were stabilized by catecholamines in the absence of PAP, but neither showed significant activity toward this class of compounds. This raises the possibility that certain endogenous and/or exogenous compounds, such as those that bind in the absence of PAP, may act as competitive inhibitors of SULTs by occupying the substrate binding pocket and preventing a productive PAPS binding conformation, as for SULT2A1 (Figure 2J).

### Screening and Structural Analysis Reveals Novel Mechanism of Inhibition

Excluding SULT4A1, three compounds bound to all hSULTs: adenosine 5′-(β,γ-imido) triphosphate (AMP-PNP), a non-hydrolysable ATP analog; pyridoxal 5-phosphate (PLP), a competitive inhibitor for sulfotransferases [34]; and quercetin, a potent inhibitor of SULT1A1 and SULT1E1 [35]. These compounds had been known to inhibit one or more sulfotransferases, but our data suggest that they may be universal SULT inhibitors. AMP-PNP and PLP bound only in the absence of saturating amounts of PAP, suggesting that they occupy the PAP binding site, as might be expected from their structural similarity to PAP. Quercetin, found in many fresh fruits and vegetables, is a flavonoid with anti-tumour and anti-inflammatory activities. It is possible that some of its favourable physiological effects may be related to inhibition of hSULT activity. Additional inhibitors bound to only a subset of the hSULTs, including 3,5-dibromo-4-hydroxy-benzoic acid (6,8-dichloro-4-oxo-4*H*-chromen-3-ylmethylene) hydrazide (DBHD), 3,5-dibromo-4-hydroxy-benzoic acid (6-chloro-4-oxo-4*H*-chromen-3-ylmethylene)-hydrazide (DBHM), and PCP.

**Figure 5:**
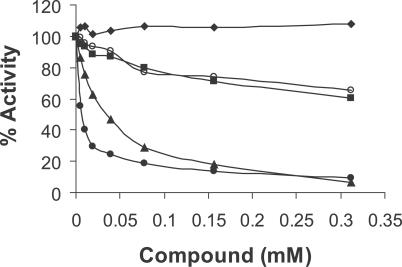
Inhibitory Effect of Isoprenaline, PLP, Quercetin, DBHD, and PCP on Sulfotransferase Activity of SULT1B1 with 1-Naphthol as Substrate Sulfotransferase activity of SULT1B1 at different concentrations of each compound (5 to 312 μM) has been assayed as described in Materials and Methods. Isoprenaline is indicated by a filled diamond, PLP by an open circle, quercetin by a filled square, DBHD by a filled triangle, and PCP by a filled circle.

The binding profiles in Table 1 raise the possibility that compounds that bind to hSULTs only in the absence of PAP may inhibit hSULT activity. In order to investigate this possibility, we assayed the activity of SULT1B1 in the presence of increasing concentrations of five compounds found in our screens. These five include known inhibitors (quercetin, DBHD, PLP, and PCP), as well as isoprenaline, which binds to SULT1B1 only in the absence of PAP and is a poor substrate for this enzyme (Tables 1 and 2). As shown in Figure 5, PCP and DBHD were strong inhibitors of SULT1B1, whereas PLP and quercetin had intermediate effects. Isoprenaline, however, had no inhibitory effect on the activity of SULT1B1 with 1-naphthol, and it was sulfonated by SULT1B1 (Table 2), indicating that not all compounds that bind in the absence of PAP are necessarily inhibitors.

**Figure 6:**
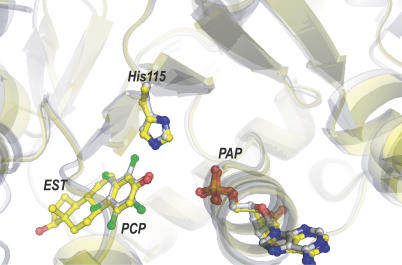
The Superimposition of the Active Sites of the SULT1E1-PAP-17β-estradiol Complex (1AQU) and the SULT1C2-PAP-PCP Complex (2GWH) The SULT1E1-PAP-17β-estradiol complex is shown in yellow, and the SULT1C2-PAP-PCP complex is shown in grey. The PCP (chlorine atoms in green) and 17β-estradiol (EST) molecules are highlighted along with the catalytic His115 and PAP. See text for discussion.

PCP is a significant environmental toxin due to its common use as a wood preservative and its use in the pulp and paper industry. Its chemical structure is related to hydroxylated metabolites of polychlorinated biphenols (OH-PCBs) whose endocrine-disruptive properties may be related to the inhibition of estradiol sulfonation by SULT1E1 [36]. The mechanism of inhibition of SULT1E1 by OH-PCBs and related compounds has been proposed to take place via both allosteric [36,37] and competitive [12,37] mechanisms. Our binding data suggest that PCP may be a competitive inhibitor of SULTs 1B1, 1C2, and 1A1 (Table 1). For 1C2 and 1A1, PAP was required for binding; and for 1B1, PCP binds much better in the presence of PAP, suggesting that PCP binds in a substrate-like conformation facilitated by PAP(S). In order to better understand the mechanism of inhibition by PCP, we determined the structure of SULT1C2 bound to PAP and PCP (Figure S3). The structure reveals that the protein undergoes a disorder–order transition upon PCP and PAP binding. Helices α4, α5, and αı5, and loops α5-α6 and α15-α16 are ordered only in the ternary complex, but not in the apo SULT1C2 structure (Figure 2B and 2H). PCP is found in the substrate binding pocket and therefore appears to be a competitive inhibitor, consistent with crystallographic analysis of SULT1E1 bound to PAP and 3,5,3′,5′-tetrachlorobiphenol [12]. Comparison of the SULT1C2-PAP-PCP and SULT1E1-PAP-estradiol structures (Figure 6) revealed two structural features that may be relevant in explaining the mechanism of PCP inhibition. In the co-crystal structures, the phenol moieties of PCP and estradiol share the same relative position and orientation, positioning the phenolic OH within hydrogen-bond distance of the catalytic histidine. This histidine is thought to be deprotonated and made catalytically competent to accept the phenolic hydrogen from estradiol, facilitating nucleophilic attack on the sulfonate of PAPS. Our structure shows that PCP appears to be in a catalytically competent conformation. However, PCP and estradiol differ dramatically in the acidity of their hydroxyl groups; the estimated pKa for estradiol is approximately 15, whereas that for PCP is 4.5. Thus, although PCP appears to bind in a catalytically competent conformation, the phenolic oxygen of PCP may be too weak a nucleophile to attack the sulfonate of PAPS.

The inhibitory effects of OH-PCBs and PCP have been interpreted previously in terms of variations in the bound conformation of the halogenated compounds relative to that of estradiol [12,36]. Although steric and conformational factors clearly play a role, our structure of SULT1C2 with PCP and PAP suggests a key role for the electronic nature of the halogenated phenols. We examined the calculated pKa values for the series of 21 4-hydroxyl–substituted PCBs for which Kester et al. [36] reported 50% inhibitory concentration (IC_50_) values. The results show a strong correlation between the calculated acidity of these phenols with IC_50_ values, with the most acidic compounds having the strongest inhibitory effect (Figure S4).

### SULT4A1 Is an Atypical SULT with an Atypical Structure

**Figure 7:**
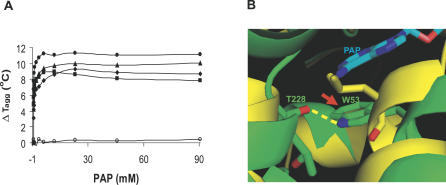
SULT4A1 Does Not Bind PAP (A) Thermal aggregation temperatures for SULT1B1 (filled circle), SULT1C3 (filled triangle), SULT1C1 (filled diamond), SULT1C2 (filled square), and SULT4A1 (open circle) as a function of PAP concentration. PAP provides no stabilizing effect to SULT4A1. (B) Superimposition of the PAP binding site of SULT1C1 (green) and SULT4A1 (yellow). A conserved Trp residue (W53 of SULT1C1; green ring in the middle of figure, indicated by a red arrow) normally forms π-π stacking interactions with the adenine ring of PAP (blue) and hydrogen bonds to a neighbouring conserved Thr (T228 in SULT1C1). SULT4A1 is the only SULT to have a Leu instead of Trp at this position. The Leu residue cannot make stabilizing interactions with Thr or the adenine ring, and its position would cause a severe steric clash with PAP and PAPS.

Under the conditions of our screens, PAP bound to all hSULTs except for SULT4A1. In order to rule out the possibility that SULT4A1 simply has a much weaker affinity for PAP, we performed titration experiments for several of the proteins with increasing concentrations of PAP ranging from 90 μM to 90 mM (Figure 7A). SULT1C1, SULT1C2, SULT1C3, and SULT1B1 showed similar saturation binding curves, reaching saturation at about 100 μM. However, PAP when added at concentrations as high as 90 mM did not stabilize SULT4A1. SULT4A1 is one of the hSULTs that is most divergent in sequence, and examination of the binding pocket revealed two significant differences that are predicted to affect PAP binding. First, a Trp in α3 that is conserved in all other hSULTs and stacks with the adenine ring of PAPS (Trp53 in SULT1A1) is replaced with a Leu in SULT4A1 (Figure 3). Second, the magenta PAP binding loop is much shorter in SULT4A1 than in the other hSULTs, and lacks the conserved Lys residue that separates the key PAPS binding residues Arg258 and Gly260 (SULT1A1 numbering), which results in these residues being out of register. Taken together, SULT4A1 has a slightly smaller PAPS binding pocket that is predicted to be unable to accommodate the cofactor. Interestingly, some residual electron density was observed in the PAP binding pocket of recombinant SULT4A1. Presumably this derives from a bound small molecule that was co-crystallized, although we were not able to identify it either by modelling atoms into the electron density or using mass spectrometry.

To test the possibility that SULT4A1 might use an alternate sulfonate donor, we tested several potential alternates such as adenosine phosphosulfate, 4-nitrocatechol sulfate, 4-acetylphenyl sulfate, estrone 3-sulfate, indoxyl sulfate, and 4-methylumbelliferyl sulfate. None of these compounds stabilized SULT4A1 against thermal aggregation (unpublished data). These data strongly suggest that SULT4A1 may not have significant catalytic activity in vivo. Indeed, although very weak activity has been reported in one case [38], other groups have failed to observe activity for human SULT4A1 [5,39]. Significantly, this protein, which is expressed primarily in the brain, binds to 2-hydroxylestradiol, thyroid hormone, T4 (3,3′,5,5′-tetraiodo-L-thyronine), and the catecholamines norepinephrine, epinephrine, and isoprenaline (but not dopamine), suggesting that SULT4A1 may modulate the bioactivity of these compounds via a mechanism distinct from sulfonation. Of note, SULT4A1 did not bind any simple phenolic compounds under the conditions tested here.

### Binding Profiles Suggest Alternative Classifications of hSULTs

**Figure 8:**
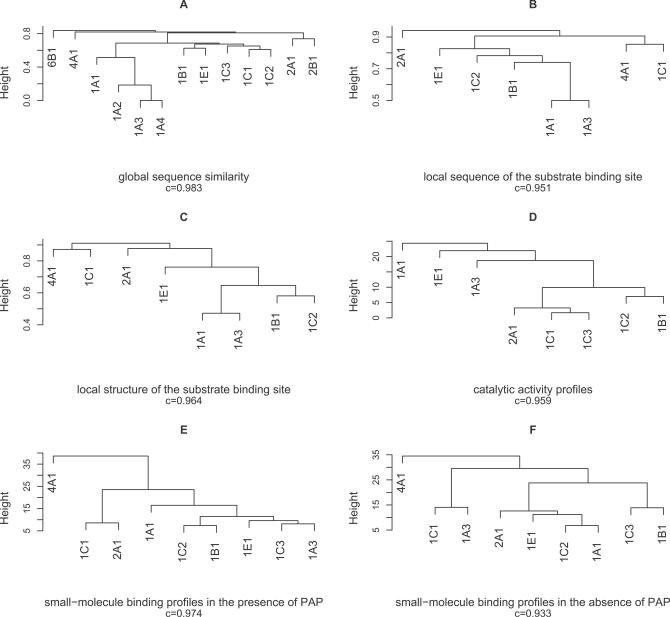
Average Linkage Hierarchical Clustering of SULTs Clustering is shown according to (A) global sequence similarity, (B) local sequence of the substrate binding site, (C) local structure of the substrate binding site, (D) catalytic activity profiles, (E) small molecule binding profiles in the presence of PAP, and (F) small molecule binding profiles in the absence of PAP. The value under each clustering (c) is the correlation between the original data matrix and the cophenetic matrix—a matrix whose elements represent the height at which these elements first meet in the tree. The higher this correlation, the more accurate the tree represents the original data. See Materials and Methods for further details.

Examination of the chemical fingerprints reflected in Tables 1 and 2 suggests that subsets of the hSULTs can be clustered based on the chemical properties of the compounds that they bind—relationships that are not evident from global sequence comparison. To explore alternative activity or structure-based classifications of hSULTs in more detail, we performed average hierarchical clustering on the experimental data in an attempt to identify correlations between the *local* sequence or structural features of the substrate binding pockets and activity profiles among the hSULTs. Figure 8 shows the clustering of similarity matrices for each parameter, viewed using trees. Considering only global sequence similarity, the hSULTs cluster according to their nomenclature and phylogenetic relationships (Figure 8A). Considering only the nine proteins for which we have binding or enzymatic data, SULTs 1A1 and 1A3 are most closely related, with a global sequence identity of 95%. The three SULT1C proteins cluster with average sequence identities close to 55%, as do SULT1E1 and 1B1. SULT2, −4, and −6 subfamilies are relative outliers with sequence identities to all other SULTs considered here around 35% or less. It is well known that related enzymes with sequence identities below 40% have often evolved to have different substrate specificities [40], and therefore, we would expect most of the SULTs to sulfonate different substrates, except perhaps 1A1 and 1A3. In keeping with this concept, the closely related SULTs 1A1 and 1A3 are clearly the most similar within the substrate binding site, as measured by both local sequence and structure comparisons (Figure 8B and 8C). However, the clustering of the other more distantly related SULTs is sufficiently different at the level of local sequence and structure, such that the SULT1C proteins are no longer clustered together and the outliers are different. These comparisons show that the local sequence and structures of the substrate binding sites do not correspond to the global sequence relationships.

**Figure 9:**
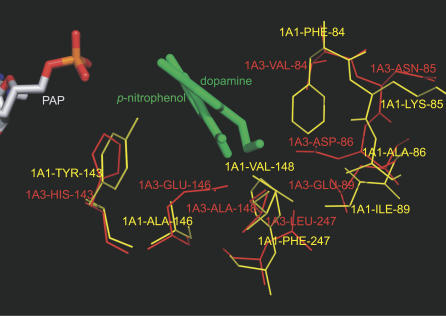
Binding Site Comparison of SULT1A1 Bound to PAP and *p*-Nitrophenol (1LS6) and SULT1A3 Bound to PAP and Dopamine (2A3R) The side chains of the eight binding-site residues that differ between these two proteins are shown in yellow (SULT1A1) and red (SULT1A3).

These results also illustrate why members of the same subfamily do not metabolize the same classes of compounds. Although the clustering of hSULTs presented here is likely influenced by the limited subset of compounds used in our binding and activity assays, the results provide an initial view of the family-wide activity-based classifications. The trees that cluster the eight or nine proteins according to their binding and activity profiles (Figure 8D–8F) show a remarkably different clustering from the sequence- and structure-based trees. First, 1A3 and 1A1 no longer cluster together, despite their strong global sequence similarity (95% identity). Inspection of Table 1 shows that 1A1 strongly binds most of the phenols and acidic compounds, once PAP is bound, whereas 1A3 shows absolutely no binding of these substrates. These SULTs are also distinguished by their differential reactivity toward catecholamines, with SULT1A3 able to bind dopamine and having higher relative activity toward catecholamines compared to phenols, as previously noted [28]. Comparison of the residues in the substrate binding loops of SULT1A1 and SULT1A3 revealed that all residues are identical (and in identical positions in the structures) except for the eight residues shown in Figure 9. These changes map to two loops (residues 84–89 and 143–148) and residue 247 (SULT1A1 numbering). Importantly, SULT1A3, which does not bind acidic compounds, has acidic instead of hydrophobic residues at three of these positions. The net result is a much more negatively charged pocket for SULT1A3 compared to SULT1A1. This difference would disfavour binding of compounds with a net negative charge to SULT1A3 and favour interactions with the amino group of catecholamines. Thus, the strong local sequence and structure similarities in 1A1 and 1A3 are manifested in their similar ability to bind similar inhibitors and sulfonate catecholamines (as a class), but small, local sequence changes in the substrate binding site have enhanced the ability of SULT1A3 to bind catecholamines such as dopamine [28], and completely changed its ability to bind acidic compounds. The influence of residue 146 on the specificity of SULT1A1 compared to SULT1A3 (Ala vs. Glu) has been previously noted [28]; however, the results presented here suggest that additional differences in the binding loops also contribute to specificity.

SULT1B1 and 1C2 cluster together in the trees of local structure, activity, and binding in the presence of PAP. These groupings reflect their common ability to bind acidic compounds (and the acidic phenols PCP and 2,6-dichloro-4-nitrophenol), with promiscuous activity profiles toward phenols. Thus, SULT1B1 and 1C2 appear to be much more closely related in terms of structure and activity than their global sequences would indicate.

Finally, Figure 8F shows that the clustering of hSULTs again differs when considering the binding of compounds in the absence of PAP. In this case, none of the previously noted similarities is evident. Many of the compounds in Table 1 are inhibitors of hSULT activity. The differential clustering in the absence of PAP may reflect possible unrelated configurations of binding-site residues when bound to these compounds or the tendency for more disorder in the absence of PAP. The clustering of both binding profiles (Figure 8E and 8F) places SULT4A1 as the furthest outlier, analogous to its position in the global sequence comparison. Although this is consistent with the inability of SULT4A1 to bind PAP and catalyze sulfonation, it is interesting to note that this is not due to a radical difference in local binding-site sequence or structure, because SULT4A1 clusters with SULT1C1 when considering these local factors (Figure 8B and 8C). As noted above, apparently small differences of one to two residues in the PAP binding site are likely responsible for this behaviour.

## Discussion

An important challenge in structural biology, chemical biology, and drug discovery is to relate changes in local sequence and structure to the binding and activity profiles of homologous enzymes in order to better predict or explain substrate and inhibitor specificity within an enzyme family. In the case of the hSULTs, this is particularly desirable in order to predict the fate of xenobiotics, hormones, and drug candidates in humans. Like other phase II detoxification enzymes, hSULTs are known to have broad and overlapping substrate specificities. We and others have shown that this promiscuity derives from the considerable flexibility or plasticity of the hSULT binding sites and therefore, a full understanding of specificity will require multiple three-dimensional structures for each hSULT in complex with substrates and inhibitors, as well as knowledge of the full spectrum of small molecules that bind in both productive and non-productive conformations. Our structural and chemical profiling data prepare the foundation for such detailed studies.

Here we also reveal a previously unrecognized structural role for the cofactor PAP(S); namely priming of the often disordered substrate binding loops for interaction with substrates. The “magenta loop” (Figure 2), which contributes to both PAPS and substrate binding pockets, can also, in some instances, adopt an inactive conformation and may explain substrate-induced inhibition and/or inhibition by compounds that bind in the absence of PAPS, as well as by known inhibitors. The flexibility of the substrate binding loops in hSULTs likely contributes to the wide repertoire of compounds that can be accommodated in the substrate binding pocket, only a subset of which lead to productive sulfonation.

Our results provide insight into mechanisms of inhibition of hSULTs. In addition to structural mechanisms of substrate inhibition (above), we identified three compounds (PLP, AMP-PNP, and quercetin) that appear to be broad-spectrum hSULT inhibitors, and may also inhibit other non-cytosolic sulfotransferases. We have also provided insight into how PCP and possibly other polychlorinated phenolic compounds can inhibit hSULTs. These compounds, which are known endocrine disruptors, appear to bind in a manner very similar to other productive substrates, but are unreactive, at least in part, due to their weakly acidic properties.

Our analyses have also provided insight into the functions of the less well-characterized SULT1C subfamily and SULT4A1. We have identified a number of novel substrates, inhibitors, and compounds that bind to these SULTs in the absence of PAP. These data combined with activity assays revealed that SULT1C3 can bind catecholamines and phenolic compounds, but only the latter are substrates. SULT4A1 is inactive as an enzyme in our experiments, likely due to its inability to bind PAPS or other sulfonate donors. This orphan SULT likely has an important function in the brain, nevertheless, because it is highly conserved and binds well to the neurotransmitters epinephrine and norepinephrine.

The approach outlined here in which simple, medium throughput binding and activity screens can be used to profile properties of purified enzymes has proven extremely useful for identification of novel substrates and analysis of specificity across a human protein family. We demonstrate that the relationship between sequence/structure and function within this small family is remarkably complex, and differences in activity can reflect just a few amino acid changes at critical locations within the protein's active site. Global sequence/structure comparisons provide good clues for broad functional classification, but cannot simply define an enzyme's cognate substrate or class of substrates. For the sulfotransferases, which have a large and flexible binding site, it is clearly necessary to perform much more detailed studies to understand both the binding and catalytic activities in terms of local structure. The actual cellular activity of hSULTs will depend on the spectrum of compounds available to a given enzyme and their relative concentrations. The tissue-specific and developmental variation in both hSULT expression and the cellular milieu of small molecules complicates further attempts to *predict* activities. Ultimately, detailed enzymatic characterization of all purified hSULTs, as well as cellular assays, will be needed to fully understand this family. The data presented here form a basis for further detailed biochemical and structural studies of both active and inactive enzyme–small molecule complexes in order to fully understand the role of hSULTs in the metabolic fate of endogenous substrates, as well as drugs and toxic compounds.

## Materials and Methods

### Protein purification and crystallization.

The SULT1B1, SULT1C1, SULT1C2, SULT1C3, and SULT4A1 genes were amplified by PCR from the Mammalian Gene Collection clones and subcloned into a modified pET28a-LIC vector. Expression and purification of recombinant proteins was as described by Dombrovski et al. [17]. Purified recombinant proteins contained an additional Gly-Ser dipeptide at the N-terminus. Additional details are provided at http://www.sgc.utoronto.ca in the structure gallery for each protein.

Purified SULT1B1, SULT1C1, and SULT1C3 were crystallized in the presence of 2 mM PAP using the hanging drop method at 20 °C by mixing: for SULT1B1—2 μl of the protein solution with 2 μl of the reservoir solution containing 0.1 M Bis-Tris (pH 6.5), 0.2 M ammonium sulfate. and 16%–20% polyethylene glycol 4000; for SULT1C1—2 μl of the protein solution with 2 μl of the reservoir solution containing 0.1 M K_2_HPO_4_ and 12%–16% polyethylene glycol 3350; and for SULT1C3—2 μl of the protein solution with 2 μl of the reservoir solution containing 18% polyethylene glycol 3350, 0.2 M ammonium formate, 0.1 M Bis-Tris (pH 6.5). To obtain crystals of SULT1C2-PAP-PCP ternary complex, 10 mg/ml of purified SULT1C2 was mixed with 2 mM PAP and 2 mM PCP in 20 mM MES-NaOH buffer (pH 6.5), and incubated on ice for 30 min. SULT1C2-PAP-PCP complex was crystallized using the sitting drop method at 20 °C by mixing 0.8 μl of the protein-cofactor-inhibitor mix with 0.8 μl of the reservoir solution containing 25% polyethylene glycol 3350, 0.2 M lithium sulfate, 0.1 M Bis-Tris (pH 6.5). SULT4A1 and SULT1C2 crystals were obtained by using the hanging drop method at 20 °C by mixing 2 μl of the protein solution with 2 μl of the reservoir solution containing 20% polyethylene glycol 4000, 0.2 M ammonium tartrate, and 14%–20% polyethylene glycol 3350, 0.2 M lithium citrate, 0.1 M sodium citrate (pH 4.6), respectively.

### Chemical library preparation.

A library of 90 compounds was created for screening sulfotransferases. These compounds were known substrates, products, and inhibitors of sulfotransferases, their analogs, and compounds with high similarity to known inhibitors identified from the literature and public databases (http://www.rcsb.org and http://www.brenda.uni-koeln.de). Certain substrates, such as controlled substances, were not included, and some additional compounds were selected through chemical similarity to known SULT substrates and inhibitors using the ChemNavigator search engine (http://www.chemnavigator.com/). The compounds were dissolved in 100% DMSO at 100 mM concentration and subsequently diluted stepwise to 10 mM and 1 mM in Hepes buffer (100 mM Hepes, 150 mM NaCl [pH 7.5]). The full list of compounds in the library is included in Table S2.

### Ligand binding screens.

Screening for ligand binding was performed in 50-μl volume with a final concentration of 1 mM of compound per well, in 384-well plates. The concentration of protein was the same for all wells at 0.4 mg/ml. Ligand binding was detected by monitoring the increase in thermostability of proteins in the presence of ligands. Protein thermostability at pH 7.5 was studied using StarGazer technology that monitors protein stability by its aggregation properties [20,21]. Protein samples at 0.4 mg/ml were heated from 27 °C to 80 °C at the rate of 0.5 °C per min in clear-bottom 384-well plates (Nunc, http://www.nuncbrand.com/) in 50 μl of 100 mM Hepes (pH 7.5) and 150 mM NaCl. Protein aggregation was monitored by capturing images of scattered light every 30 s with a CCD (Charge-Coupled Device) camera. The pixel intensities in a pre-selected region of each well were integrated to generate a value representative of the total amount of scattered light in that region. These total intensities were then plotted against temperature for each sample well and fit to the Boltzman equation by nonlinear regression. The point of inflection of each “denaturation” curve was identified as *T*_agg_ (aggregation temperature). The increase in stability of the protein in the presence of a ligand is shown as Δ*T*_agg_.

### Sulfotransferase activity screens.

Enzyme assays were performed using a HPLC-based method that we developed for sulfotransferase activity assay by modifying the protocol previously used to monitor ADP production and ATP hydrolysis by a purified bacterial ATPase [41]. SULTs at 1–5 μM were assayed in the presence of 0.1 to 0.5 mM PAPS and different concentrations of each substrate in 100 mM Hepes (pH 7.5) by incubating the reaction at 37 °C for a period of time from 15 to 120 min depending on how fast PAPS was converted to PAP. The K_m_ values for characterized sulfotransferases are in the range of nanomolar to millimolar concentrations [24,42], with a significant variation in catalytic efficiency and substrate specificity. Based on these observations and considering possible substrate inhibition [19,43], we tested all sulfotransferases at substrate concentrations of 10, 25, and 100 μM. The reactions were stopped by adding two volumes of urea (final concentration of 5.3 M), and the mixture was filtered through a 5-kDa molecular weight cutoff Amicon Ultrafree-MC filter (Millipore, Bedford, Massachusetts, United States) to remove the protein. The ratio of PAP and PAPS was determined after separating them on HPLC using a 4.5 mm × 50 mm WP QUAT, a strong ion-exchange column (J. T. Baker, Phillipsburg, New Jersey, United States), using a gradient of triethylamine bicarbonate from 20 to 500 mM applied at 2 ml/min for 7 min. The progress of the reaction was monitored by reading the absorbance at 259 nm, and the amount of PAP produced was determined by integration of the resolved peaks using the HPLC software (Waters, http://www.waters.com/). All values in Table 2 were corrected for the rate of conversion of PAPS to PAP in the presence of enzyme, but no substrate. This background activity is reported for each SULT in the last row of Table 2, and the values are the average of three independent measurements.

### Sequence, structure, and data clustering analysis.

Sequences used to generate Figures 3 and 8 are as follows: SULT1A1, SULT1A2, SULT1A3, SULT1A4, SULT1B1, SULT1C1, SULT1C2, SULT1C3, SULT1E1, SULT2A1, SULT2B1, SULT4A1, and SULT6B1. We created a multiple sequence alignment of the above-mentioned sequences using HMMer [44] and the pfam [45] Sulfotransferase_1 (PF00685.15) Hidden Markov Model. Sequence similarity is measured using the Tanimoto coefficient of residues in common in the HMM-based alignment. The calculation of local sequence similarities involves the detection of binding site residues [46], and their subsequent mapping onto the HMM-based alignment. Cofactor (PAP/PAPS) binding residues were also mapped onto the alignment and excluded from all pairwise comparisons and similarity calculations. Substrate binding-site structural similarities were detected using a two-stage graph-matching process providing a one-to-one chemical and spatial correspondence between atoms in clefts. The method considers all non-hydrogen atoms and can use large sets of atoms as input allowing larger, over-predicted and apo-form binding sites to be analyzed. In the first stage, the two clefts are superimposed [47] via the detection of the largest clique [48] in a C_α_ association graph corresponding to the largest subset of identical residues in equivalent spatial locations. The first stage is used to constrain the construction of the second-stage all-atom association graph. The second-stage graph-matching results in the detection of the largest subset of heavy atoms of equivalent atoms types [49], and spatial positions. Pairwise local structural similarity was calculated as a Tanimoto coefficient based on the size of the largest clique in the second graph-matching stage. Dissimilarity matrices were derived from the similarity measures described above. Pairwise experimental catalytic and binding-profile dissimilarity matrices were calculated as the L_2_ distance of the vectors with the corresponding experimental measurements. Hierarchical clustering was used to create the clustering trees shown in Figure 8. The correlation between the cophenetic matrix and the original dissimilarity matrix was used to choose the linkage method that results in the most accurate representation of the original data [50]. Average linkage was found to be the clustering method of choice in all instances.

### pKa calculations of polyphenol hydroxyl moiety.

To assess the extent to which the acidity of the hydroxyl moiety of hydroxylated polychlorinated biphenols is related to their inhibitory strength, we computed the pKa values of the 4-hydroxyl group for a series of hydroxylated PCB analogs. This group of compounds and their inhibitory effect on SULT1E1 were previously reported [36]. To this end, we used the pKa calculator in the PC stand-alone version of the ACD (http://www.acdlabs.com) suite of programs. Two clusters of compounds, namely 4-OH-(2,3,4,5,6)Cl and 4-OH-(3,5)Cl, are identified as outliers upon computing a linear regression on the relationship (*R* ^2^ = 0.57).

## Supporting Information

Figure S1 Stereo Images of Representative Model-Phased Electron Density (3.1 MB DOC)

Figure S2 Additional Structures of Human SULTs from the Protein Data Bank (1.3 MB DOC)

Figure S3 Stereo Image of Model-Phased 2Fo-Fc and Anomalous Difference Fourier Maps for the SULT1C2:PAP:PCP Complex (700 KB DOC)

Figure S4 Correlation between Computed pKa Values and Published IC_50_ Values of Hydroxylated Polychlorinated Biphenyls on hSULT1E1 (46 KB DOC)

Table S1 Data Collection and Refinement Statistics (95 KB DOC)

Table S2 List of 90 Compounds in SULT Compound Library (102 KB DOC)

### Accession Numbers

The National Center for Biotechnology Information (http://www.ncbi.nlm.nih.gov) Reference Sequence (RefSeq) accession numbers for the proteins referred to in this paper are SULT1A1 (NP_803880), SULT1A2 (NP_001045), SULT1A3 (NP_003157), SULT1A4 (NP_001017389), SULT1B1 (NP_055280), SULT1C1 (NP_789795), SULT1C2 (NP_006579), SULT1C3 (NP_001008743), SULT1E1 (NP_005411), SULT2A1 (NP_003158), SULT2B1 (NP_814444), SULT4A1 (NP_055166), and SULT6B1 (NP_001027549).

The Mammalian Gene Collection (http://mgc.nci.nih.gov) clones discussed in this paper are SULT1B1 (gi: 29550928), SULT1C1 (gi: 4507305), SULT1C2 (gi: 28830308), SULT1C3 (gi: 56847626), and SULT4A1 (gi: 7657633).

The Protein Data Bank (http://www.pdb.org) IDs for the proteins discussed in this paper are SULT1A3 (1CJM), SULT1C2 (2AD1), SULT4A1 (1ZD1), SULT1C1 with PAP (2ETG), SULT1B1 with PAP (1XV1), SULT1C3 with PAP (2H8K), SULT1A1 with PAP and *p*-nitrophenol (1LS6), SULT2B1b with PAP and pregnenolone (1Q20), SULT1E1 with PAP and 3,5,3′,5′-tetrachloro-biphenyl-4,4′-diol (1G3M), SULT2A1 with DHEA (1J99), SULT2A1 with PAP (1EFH), SULT1C2 with PAP and PCP (2GWH), SULT1A3 with PAP and dopamine (2A3R), SULT1A1 with PAP and estradiol (2D06), SULT2A1 with androsterone (1OV4), SULT2B1a with PAP and 2-[*N*-cyclohexylamino]ethane sulfonic acid (1Q1Q), and SULT2B1b with PAP and DHEA (1Q22).

## Abbreviations

DHEA: dehydroepiandrosterone
HPLC: high-performance liquid chromatography
hSULT: human cytosolic sulfotransferase
PAP(S): PAPS or PAP
PCB: polychlorinated biphenyl
PCP: pentachlorophenol
SULT: cytosolic sulfotransferase
