# Correction: Structural and Chemical Profiling of the Human Cytosolic Sulfotransferases

**DOI:** 10.1371/journal.pbio.0050165

**Published:** 2007-06-12

**Authors:** Abdellah Allali-Hassani, Wang Pan, Ludmila Dombrovski, Rafi Najmanovich, Wolfram Tempel, Aiping Dong, Peter Loppnau, Fernando Martin, Janet Thornton, Aled Edwards, Alexey Bochkarev, Alexander Plotnikov, Masoud Vedadi, Cheryl Arrowsmith

In *PLoS Biology*, volume 5, issue 5: doi: 10.1371/journal.pbio.0050097

The ninth author's name was incorrectly given as Janet Thonton; it should be Janet Thornton.

