# Supplementary material for: Structural and Chemical Profiling of the Human Cytosolic Sulfotransferases
Source: PLoS Biol. 2007 Apr 10;5(5):e97. doi: 10.1371/journal.pbio.0050097 (PMC1847840; doi:10.1371/journal.pbio.0050097)
Supplement: Supplementary file 1 [file pbio.0050097.sg001.doc]

A	B	C

**Supporting Figure 1 –** Stereo images of representative model-phased electron density for coordinates of SULT1C3 (A, PDB ID 2H8K), SULT4A1 (B, PDB ID 1ZD1) and SULT1C2 (C, PDB ID 2AD1).
