# Supplementary material for: Structural and Chemical Profiling of the Human Cytosolic Sulfotransferases
Source: PLoS Biol. 2007 Apr 10;5(5):e97. doi: 10.1371/journal.pbio.0050097 (PMC1847840; doi:10.1371/journal.pbio.0050097)
Supplement: Supplementary file 2 [file pbio.0050097.sg002.doc]

2B1b

1A1

1A3

2B1a

2B1b

**D**

**A**

**B**

**E**

**F**

2B1b

**C**

**Supporting Figure 2 –** Additional structures of human SULTs from the PDB: (A) SULT1A1 with PAP and estradiol (2D06), (B) SULT1A3 with PAP and dopamine (2A3R), (C) SULT2B1b with PAP and pregenolone (1Q20), (D) SULT2B1b with PAP (1Q1Z), (E) SULT2B1a with PAP and 2-[N-cyclohexylamino] ethane sulfonic acid (1Q1Q), and (F) SULT2B1b with PAP and DHEA (1Q22). For more details see Figure 2 legend.
