# Supplementary material for: Structural and Chemical Profiling of the Human Cytosolic Sulfotransferases
Source: PLoS Biol. 2007 Apr 10;5(5):e97. doi: 10.1371/journal.pbio.0050097 (PMC1847840; doi:10.1371/journal.pbio.0050097)
Supplement: Supplementary file 3 [file pbio.0050097.sg003.doc]

**Supporting Figure 3** – Stereo image of model-phased 2Fo-Fc (1.8Å resolution, 1.4σ, blue) and anomalous difference Fourier (2.5Å, 3σ, red) maps for the SULT1C2:PAP:PCP complex (PDB ID 2GWH).
