# Supplementary material for: Structural and Chemical Profiling of the Human Cytosolic Sulfotransferases
Source: PLoS Biol. 2007 Apr 10;5(5):e97. doi: 10.1371/journal.pbio.0050097 (PMC1847840; doi:10.1371/journal.pbio.0050097)
Supplement: Supplementary file 4 [file pbio.0050097.sg004.doc]

**Supporting Figure 4 –**  Correlation between computed pKa values and published IC_50_ values of hydroxylated polychlorinated biphenyls on hSULT1E1. Compounds **2-22** of Kester et. al[1] comprise a series of 4-OH substituted polychlorinated biphenyls and are plotted above. All data points were included in the regression analysis. The “outliers” from the regression line correspond to **4-OH-2,3,5,6,**2’,4’,5’-heptachlorobiphenyl (highlighted in green), and **4-OH-3,5**,3’,4’-tetrachlorobiphenyl, **4-OH-3,5**,3’,5’-tetrachlorobiphenyl, **4-OH-3,5,**2’,3’,4’-pentachlorobiphenyl, **4-OH-3,5,**3’,4’,5’-pentachlorobiphenyl (highlighted in orange). The latter series (orange) are particularly potent inhibitory compounds and also have a linear relationship between LogIC_50_ vs computed pKa within this structural series.

**Reference:**

1. Kester MH, Bulduk S, Tibboel D, Meinl W, Glatt H, et al. (2000) Potent inhibition of estrogen sulfotransferase by hydroxylated PCB metabolites: a novel pathway explaining the estrogenic activity of PCBs. Endocrinology 141: 1897-1900.
