# Supplementary material for: Structural and Chemical Profiling of the Human Cytosolic Sulfotransferases
Source: PLoS Biol. 2007 Apr 10;5(5):e97. doi: 10.1371/journal.pbio.0050097 (PMC1847840; doi:10.1371/journal.pbio.0050097)
Supplement: Supplementary file 5 [file pbio.0050097.st001.doc]

**Supporting Table 1. Data collection and refinement statistics**.

Reflections were indexed, integrated and scaled with the HKL2000 suite[1]. Structures were solved by molecular replacement using MOLREP[2] and PHASER[3]. Except for SULT1C3, an initial model was build automatically by ARP/wARP[4]. Restrained coordinate and temperature factor refinement in CNS[5] and REFMAC5[6] and manual rebuilding in O[7] or COOT[8] were iterated for several cycles.

|  | SULT1C2 | SULT1C3-PAP | SULT4A1 | SULT1C2-PAP-PCP |
| --- | --- | --- | --- | --- |
| **PDB code** | 2AD1 | 2H8K | 1ZD1 | 2GWH |
|  |  |  |  |  |
| **Data collection** |  |  |  |  |
| X-ray source | Rigaku FR-E | APS 17ID | Rigaku FR-E | Rigaku FR-E |
| Wavelength (Å) | 1.5418 | 1.0000 | 1.5418 | 1.5418 |
| Space group | P3_2_21 | C222_1_ | C2 | I422 |
| Cell dimensions |  |  |  |  |
| *a*, *b*, *c* (Å) | 58.82, 58.82, 189.29 | 87.16, 108.72, 136.92 | 98.94, 74.20, 85.83 | 135.22, 135.22, 158.02 |
| α, β, γ (°) | 90, 90, 120 | 90, 90, 90 | 90, 105.52, 90 | 90, 90, 90 |
| Resolution (Å)* | 50.00-2.00 (2.07-2.00) | 100-3.00 (3.11-3.00) | 50.00-2.24 (2.28-2.24) | 30.00-1.80 (1.85-1.80) |
| *R*_sym_ or *R*_merge_ | 0.059 (0.767) | 0.149 (0.651) | 0.087 (0.600) | 0.111 (0.000) |
| *I* / σ*I* | 14.6 (2.9) | 10.5 (1.1) | 9.2 (2.6) | 26.4 (1.9) |
| Completeness (%) | 93.7 (72.5) | 98.4 (87.9) | 99.1 (97.6) | 99.9 (99.5) |
| Redundancy | 10.7 (9.5) | 5.7 (4.0) | 3.8 (3.6) | 14.0 (10.8) |
|  |  |  |  |  |
| **Molecular replacement** |  |  |  |  |
| Search model PDB code | 1ZHE[9] | 2AD1 | 1CJM[10] | 2AD1 |
|  |  |  |  |  |
| **Refinement** |  |  |  |  |
| Resolution (Å) | 50.00-2.00 | 30.00-3.20 | 50.00-2.24 | 30.00-1.80 |
| No. reflections | 23766 | 10487 | 27265 | 65377 |
| *R*_work_ / *R*_free_ | 0.218/0.262 | 0.292/0.343 | 0.186/0.262 | 0.191/0.219 |
| No. atoms | 2204 | 3616 | 4326 | 5131 |
| Protein | 2051 | 3562 | 4098 | 4726 |
| Ligand/ion | N/A | 54 | 6 | 90 |
| Water | 153 | N/A | 222 | 315 |
| *B*-factors (Å^2^) | 42.75 | 65.88 | 37.44 | 25.24 |
| Protein | 42.49 | 65.97 | 37.42 | 25.01 |
| Ligand/ion | N/A | 60.13 | 58.34 | 26.30 |
| Water | 46.16 | N/A | 37.18 | 28.60 |
| R.m.s deviations |  |  |  |  |
| Bond lengths (Å) | 0.021 | 0.016 | 0.025 | 0.016 |
| Bond angles (°) | 1.7 | 1.4 | 2.0 | 1.3 |
| Ramachandran plot  % residues(2) |  |  |  |  |
| Favored | 90.6 | 82.8 | 91.7 | 91.3 |
| Additional allowed | 8.9 | 15.8 | 6.2 | 8.7 |
| Generously allowed | 0.0 | 1.4 | 1.8 | 0.0 |
| Disallowed | 0.5 | 0.0 | 0.2 | 0.0 |

*Highest resolution shell is shown in parenthesis.

**References**:

1. Otwinowski Z, Minor W (1997) Processing of X-ray diffraction data collected in oscillation mode. Macromolecular Crysallography, Pt a 276: 307-326.

2. Vagin A, Teplyakov A (1997) MOLREP: An automated program for molecular replacement. Journal of Applied Crystallography 30: 1022-1025.

3. McCoy AJ, Grosse-Kunstleve RW, Storoni LC, Read RJ (2005) Likelihood-enhanced fast translation functions. Acta Crystallographica Section D - Biological Crystallography 61: 458-464.

4. Perrakis A, Morris R, Lamzin VS (1999) Automated protein model building combined with iterative structure refinement. Nature Structural Biology 6: 458-463.

5. Brunger AT, Adams PD, Clore GM, Delano WL, Gros P, et al. (1998) Crystallography & MNR system: A new software suite for macromolecular structure determination. Acta Crystallographica Section D - Biological Crystallography 54 pt 5: 905-921.

6. Murshudow GN, Vagin AA, Dodson EJ (1997) Refinement of macromolecular structures by the maximum-likelihood method. Acta Crystallographica Section D - Biological Crystallography 53: 240-255.

7. Jones TA, Zou JY, Cowan SW, Kjeldgaard M (1991) Improved methods for building protein models in the electron-density maps and the location of errors in these models. Acta Crystallographica Section A 47: 110-119.

8. Emsley P, Cowtan K (2004) Coot: Model-building tools for molecular graphics. Acta Crystallographica Section D - Biological Crystallography 60: 2126-2132.

9. Dombrovski L, Dong A, Bochkarev A, Plotnikov AN (2006) Crystal structures of human sulfotransferases SULT1B1 and SULT1C1 complexed with the cofactor product adenosine-3'- 5'-diphosphate (PAP). Proteins 64: 1091-1094.

10. Bidwell LM, McManus ME, Gaedigk A, Kakuta Y, Negishi M, et al. (1999) Crystal structure of human catecholamine sulfotransferase. J Mol Biol 293: 521-530.
