# Supplementary material for: Structural and Chemical Profiling of the Human Cytosolic Sulfotransferases
Source: PLoS Biol. 2007 Apr 10;5(5):e97. doi: 10.1371/journal.pbio.0050097 (PMC1847840; doi:10.1371/journal.pbio.0050097)
Supplement: Supplementary file 6 [file pbio.0050097.st002.doc]

**Supporting Table 2**. **List of 90 compounds in SULT compound library.**

**Compound**

mefenamic acid

salbutamol

R-(-)-apomorphine hydrochloride hemihydrate

salicylic acid

quercetin dihydrate

resveratrol

acetaminophen (paracetamol)

isoprenaline hydrochloride

vanillin

tyramine

*p*-cresol

4-aminophenol

(±)-epinephrine

pentachlorophenol

2,6-dinitrotoluene

2,4-dinitrotoluene

α-zearalenol

2-hydroxyestradiol

4-ethylphenol

lithocholic acid

(±)-chlorpheniramine maleate salt

4-nitrophenol

clomiphene citrate salt

adenosine-3',5'-bisphosphate

N-(iodoacetyl-aminoethyl)-5-N'-naphthylamin-1-sulfonic acid (1,5-IAEDANS)

N-bromosuccinimide

*p*-chloromercuribenzoate

2,6-dichloro-4-nitrophenol

3-hydroxyindole (1H-indol-3-ol dihydrogen phosphate ester, disodium salt)

dopamine (hydrochloride)

minoxidil

(+)-(S)-1,2,3,4-tetrahydro-1-naphthol

1-naphthol

2,3-butanedione

2-ethylphenol

2-*n*-propylphenol

2-*sec*-butylphenol

4-*n*-amylphenol (4-pentylphenol)

4-*n*-heptylphenol

3,5-dibromo-4-hydroxy-benzoic acid (6,8-dichloro-4-oxo-4H-chromen-3-ylmethylene)-hydrazide

3,5-dibromo-4-hydroxy-benzoic acid (6-chloro-4-oxo-4H-chromen-3-ylmethylene)-hydrazide

N-acetylglucosamine 6-sulfate sodium salt, mixed anomers

sucrose

D-(+) glucose

2-aminofluorene

17α-ethynylestradiol

dexamethasone

estrone

thienylbutyl isothiocyanate

thienyldecyl isothiocyanate

thienyldodecyl isothiocyanate

γ-aminobutyric acid - GABA

acetylcholine chloride

(-)-norepinephrine

histamine dihydrochloride

cytidine 5'-diphosphocholine

pyridoxal 5’-phosphate

thiamine monophosphate

niacin - vitamin B3

argininosuccinic acid disodium salt

DL-glyceraldehyde

L-ornithine monohydrochloride

L-citrulline

coenzyme B12

ADP - Na

CDP - Na

GDP - Na

UDP - Na

ATP - diNa

CTP - Na

GTP - Na

ITP - triNa

UTP - Na

AMP-PNP (adenosine 5′-(β,γ-imido)triphosphate)

GMP-PNP (5'-guanosyl-imido-triphosphate)

guanosine

T2 (3,5-diiodo-L-thyronine)

T3 (3,3′,5-triiodo-L-thyronine sodium salt)

T4 (3,3′,5,5'-tetraiodo-L-thyronine) (L-thyroxine)

dehydroisoandrosterone 3-sulfate sodium salt

2-naphthol

dATP

dGTP

dCTP

dTTP

Rp-cAMPS triethylammonium salt (Rp-Adenosine 3′,5′-cyclic monophosphorothioate triethylammonium salt hydrate)

D-(+)-glucosamine hydrochloride

D-glucosamine 6-sulfate

H-7 (1-(5-isoquinolinesulfonyl)-2-methylpiperazine)

H-7 dihydrochloride (1-(5-isoquinolinesulfonyl)-2-methylpiperazine dihydrochloride)

control 100 mM HEPES 150 mM NaCl pH 7.5 1% DMSO

control 100 mM HEPES 150 mM NaCl pH 7.5
